
JOURNAL INFORMATION
==============================
NLM Title Abbreviation: Natl Sci Rev
Journal ID: Natl Sci Rev
NLM Journal ID: 101633095
Journal ID: nsr

National Science Review

ISSN: 2095-5138
EISSN: 2053-714X

ARTICLE INFORMATION
==============================
PMCID: PMC7665658
PMID: 34676083
DOI: 10.1093/nsr/nwaa264
Article ID: nwaa264
Article version: 1
Subjects: Brief Communication, AcademicSubjects/MED00010, AcademicSubjects/SCI00010

Cold-chain food contamination as the possible origin of Covid-19 resurgence in Beijing

Pang Xinghuo Beijing Center for Disease Prevention and Control (CDC), Beijing 100013, China
Research Centre for Preventive Medicine of Beijing, Beijing 100013, China

Ren Lili NHC Key Laboratory of Systems Biology of Pathogens and Christophe Mérieux Laboratory, Institute of Pathogen Biology, Chinese Academy of Medical Sciences & Peking Union Medical College, Beijing 100730, China
Key Laboratory of Respiratory Disease Pathogenomics, Chinese Academy of Medical Sciences and Peking Union Medical College, Beijing 100730, China

Wu Shuangsheng Beijing Center for Disease Prevention and Control (CDC), Beijing 100013, China
Research Centre for Preventive Medicine of Beijing, Beijing 100013, China

Ma Wentai Beijing Institute of Genomics, Chinese Academy of Sciences, and China National Center for Bioinformation, Beijing 100101, China
University of Chinese Academy of Sciences, Beijing 100049, China

http://orcid.org/0000-0002-8826-5198 Yang Jian NHC Key Laboratory of Systems Biology of Pathogens, Institute of Pathogen Biology, Chinese Academy of Medical Sciences & Peking Union Medical College, Beijing 100730, China

Di Lin Beijing Advanced Innovation Center for Genomics (ICG), Biomedical Pioneering Innovation Center (BIOPIC), College of Chemistry, and Peking-Tsinghua Center for Life Sciences, Peking University, Beijing 100871, China

Li Jie School of Life Scences, Tsinghua-Peking Center for Life Sciences, Tsinghua University, Beijing 100084, China

Xiao Yan NHC Key Laboratory of Systems Biology of Pathogens and Christophe Mérieux Laboratory, Institute of Pathogen Biology, Chinese Academy of Medical Sciences & Peking Union Medical College, Beijing 100730, China
Key Laboratory of Respiratory Disease Pathogenomics, Chinese Academy of Medical Sciences and Peking Union Medical College, Beijing 100730, China

Kang Lu Beijing Institute of Genomics, Chinese Academy of Sciences, and China National Center for Bioinformation, Beijing 100101, China
University of Chinese Academy of Sciences, Beijing 100049, China

Du Shichang Beijing Center for Disease Prevention and Control (CDC), Beijing 100013, China
Research Centre for Preventive Medicine of Beijing, Beijing 100013, China

Du Jing Beijing Center for Disease Prevention and Control (CDC), Beijing 100013, China
Research Centre for Preventive Medicine of Beijing, Beijing 100013, China

Wang Jing Beijing Center for Disease Prevention and Control (CDC), Beijing 100013, China
Research Centre for Preventive Medicine of Beijing, Beijing 100013, China

Li Gang Beijing Center for Disease Prevention and Control (CDC), Beijing 100013, China
Research Centre for Preventive Medicine of Beijing, Beijing 100013, China

Zhai Shuguang Beijing Center for Disease Prevention and Control (CDC), Beijing 100013, China
Research Centre for Preventive Medicine of Beijing, Beijing 100013, China

Chen Lijuan Beijing Center for Disease Prevention and Control (CDC), Beijing 100013, China
Research Centre for Preventive Medicine of Beijing, Beijing 100013, China

Zhou Wenxiong Beijing Advanced Innovation Center for Genomics (ICG), Biomedical Pioneering Innovation Center (BIOPIC), College of Chemistry, and Peking-Tsinghua Center for Life Sciences, Peking University, Beijing 100871, China

Lai Shengjie WorldPop, School of Geography and Environmental Science, University of Southampton, England SO17 1BJ, UK

Gao Lei NHC Key Laboratory of Systems Biology of Pathogens, Institute of Pathogen Biology, Chinese Academy of Medical Sciences & Peking Union Medical College, Beijing 100730, China

Pan Yang pan_yang@126.com Beijing Center for Disease Prevention and Control (CDC), Beijing 100013, China
Research Centre for Preventive Medicine of Beijing, Beijing 100013, China

Wang Quanyi bjcdcxm@126.com Beijing Center for Disease Prevention and Control (CDC), Beijing 100013, China
Research Centre for Preventive Medicine of Beijing, Beijing 100013, China

Li Mingkun limk@big.ac.cn Beijing Institute of Genomics, Chinese Academy of Sciences, and China National Center for Bioinformation, Beijing 100101, China
University of Chinese Academy of Sciences, Beijing 100049, China
Center for Excellence in Animal Evolution and Genetics, Chinese Academy of Sciences, Kunming 650223, China

Wang Jianbin jianbinwang@tsinghua.edu.cn School of Life Scences, Tsinghua-Peking Center for Life Sciences, Tsinghua University, Beijing 100084, China
Beijing Advanced Innovation Center for Structural Biology (ICSB), Tsinghua University, Beijing 100084, China
Chinese Institute for Brain Research (CIBR), Beijing 102206, China

http://orcid.org/0000-0002-7297-1266 Huang Yanyi yanyi@pku.edu.cn Beijing Advanced Innovation Center for Genomics (ICG), Biomedical Pioneering Innovation Center (BIOPIC), College of Chemistry, and Peking-Tsinghua Center for Life Sciences, Peking University, Beijing 100871, China
Institute for Cell Analysis, Shenzhen Bay Laboratory, Guangdong 518132, China

Wang Jianwei wangjw28@163.com NHC Key Laboratory of Systems Biology of Pathogens and Christophe Mérieux Laboratory, Institute of Pathogen Biology, Chinese Academy of Medical Sciences & Peking Union Medical College, Beijing 100730, China
Key Laboratory of Respiratory Disease Pathogenomics, Chinese Academy of Medical Sciences and Peking Union Medical College, Beijing 100730, China

COVID-19 Field Response Group Beijing Center for Disease Prevention and Control (CDC), Beijing 100013, China
Research Centre for Preventive Medicine of Beijing, Beijing 100013, China

COVID-19 Laboratory Testing Group Beijing Center for Disease Prevention and Control (CDC), Beijing 100013, China
Research Centre for Preventive Medicine of Beijing, Beijing 100013, China

Corresponding author. E-mail: wangjw28@163.comCorresponding author. E-mail: yanyi@pku.edu.cnCorresponding author. E-mail: jianbinwang@tsinghua.edu.cnCorresponding author. E-mail: limk@big.ac.cnCorresponding author. E-mail: pan_yang@126.comCorresponding author. E-mail: bjcdcxm@126.comEqually contributed to this work.

Electronic publication date: 2020 Oct 23
Electronic Location ID: nwaa264
Copyright: © The Author(s) 2020. Published by Oxford University Press on behalf of China Science Publishing & Media Ltd.
Copyright year: 2020
License: This is an Open Access article distributed under the terms of the Creative Commons Attribution License (http://creativecommons.org/licenses/by/4.0/), which permits unrestricted reuse, distribution, and reproduction in any medium, provided the original work is properly cited.
License URL: https://creativecommons.org/licenses/by/4.0/

edited-state: accepted-manuscript
article-lifecycle: PAP

==============================
**Brief Communication**

### Cold-chain food contamination as the possible origin of Covid-19 resurgence in Beijing

Xinghuo Pang[1,2,†], Lili Ren[3,4,†], Shuangsheng Wu[1,2,†], Wentai Ma[5,6,†], JianYang[7], Lin Di[8], Jie Li[9], Yan Xiao[3,4], Lu Kang[5,6], Shichang Du[1,2], Jing Du,[1,2] Jing Wang[1,2], Gang Li[1,2], Shuguang Zhai[1,2], Lijuan Chen[1,2], Wenxiong Zhou[8], Shengjie Lai[10], Lei Gao[7], Yang Pan[1,2,*], Quanyi Wang[1,2,*], Mingkun Li[5,6,11,*], Jianbin Wang[9,12,13,*], Yanyi Huang[8,14,*], Jianwei Wang[3,4,*], COVID-19 Field Response Group[1,2], and COVID-19 Laboratory Testing Group[1,2]

[1] Beijing Center for Disease Prevention and Control (CDC), Beijing 100013, China

[2] Research Centre for Preventive Medicine of Beijing, Beijing 100013, China

[3] NHC Key Laboratory of Systems Biology of Pathogens and Christophe Mérieux Laboratory, Institute of Pathogen Biology, Chinese Academy of Medical Sciences & Peking Union Medical College, Beijing 100730, China

[4] Key Laboratory of Respiratory Disease Pathogenomics, Chinese Academy of Medical Sciences and Peking Union Medical College, Beijing 100730, China

[5] Beijing Institute of Genomics, Chinese Academy of Sciences, and China National Center for Bioinformation, Beijing 100101, China

[6] University of Chinese Academy of Sciences, Beijing 100049, China

[7] NHC Key Laboratory of Systems Biology of Pathogens, Institute of Pathogen Biology, Chinese Academy of Medical Sciences & Peking Union Medical College, Beijing 100730, China

[8] Beijing Advanced Innovation Center for Genomics (ICG), Biomedical Pioneering

ORIGINAL UNEDITED MANUSCRIPT

© The Author(s) 2020. Published by Oxford University Press on behalf of China Science Publishing & Media Ltd. This is an Open Access article distributed under the terms of the Creative Commons Attribution License (http://creativecommons.org/licenses/by/4.0/), which permits unrestricted reuse, distribution, and reproduction in any medium, provided the original work is properly cited.

Innovation Center (BIOPIC), College of Chemistry, and Peking-Tsinghua Center for Life Sciences, Peking University, Beijing 100871, China

[9] School of Life Scences, Tsinghua-Peking Center for Life Sciences, Tsinghua University, Beijing 100084, China

[10] WorldPop, School of Geography and Environmental Science, University of Southampton, England SO17 1BJ, UK

[11] Center for Excellence in Animal Evolution and Genetics, Chinese Academy of Sciences, Kunming 650223, China

[12] Beijing Advanced Innovation Center for Structural Biology (ICSB), Tsinghua University, Beijing 100084, China.

[13] Chinese Institute for Brain Research (CIBR), Beijing 102206, China

[14] Institute for Cell Analysis, Shenzhen Bay Laboratory, Guangdong 518132, China

[†] Equally contributed to this work.

* Corresponding authors. E-mails: wangjw28@163.com (J. W.), yanyi@pku.edu.cn (Y.H.), jianbinwang@tsinghua.edu.cn (Jianbin W.), limk@big.ac.cn (M. L.), pan_yang@126.com (Y. P.), bjcdcxm@126.com (Q. W.)

ORIGINAL UNEDITED MANUSCRIPT

Covid-19, caused by SARS-CoV-2 [1,2], has been contained in China through stringent non-pharmaceutical interventions. The border control and quarantine have effectively prevented the virus spread by infected travellers, but the risk of resurgence caused by other routes of introduction and transmission remains unclear, and current strategies to prevent resurgence could be flawed. Since July, SARS-CoV-2 RNA contaminations in frozen food imported from countries with ongoing epidemics have been reported in nine provinces in China [3,4]. However, there is no robust evidence of Covid-19 outbreaks initiated by environment-to-human transmission. Here we add to evidence of such transmission by investigating the recent Covid-19 resurgence in Beijing.

On June 11$^{th}$, 2020, a 52-year old man suffering from fever and cough was diagnosed with Covid-19 in Beijing, after a 56-day zero new case interval. He had no exposure history of known Covid-19 cases. On June 12$^{th}$, 112 close contacts of the index case and 242 environmental samples collected from the places that he had visited were tested by qRT-PCR. All close contacts were negative, but two environmental samples from Xinfadi Market (XFDM) were positive for SARS-CoV-2. This led to in-depth investigation to confirm the role of XFDM in virus spread. 538 employees from the booths that were close to the SARS-CoV-2-positive environmental samples were tested, and 45 were positive by qRT-PCR.

To evaluate the extent of infection spreading, a screening campaign of SARS-CoV-2 infection was implemented over the city by Beijing CDC. Between June 15$^{th}$ and July 10$^{th}$, a total of more than 10 million citizens, and 5,342 environmental samples were screened. Eventually 368 qRT-PCR positive cases were confirmed (**Figure S1A**), of which 169 (46.0%) had a history of working in XFDM. 103 (28.0%) visitors to XFDM during May 30 to June 12 were diagnosed. The remaining 96 (26.2%) patients had contact with the infected employees or visitors. These findings suggested a single outbreak source in Beijing (**Figure S1B**). Retrospective epidemiological investigation revealed the earliest symptom onset of a patient on June 4 (**Figure S1C**).

To probe the origin of the infection, we analysed the spatial distribution of infected employees in XFDM. Strikingly, 20.9% (122/584) of employees working in the basement of the XFDM trading hall (XFDM-TH) were positive for SARS-CoV-2,

ORIGINAL UNEDITED MANUSCRIPT

which is significantly higher than those of other areas in the market (1.7%, 47/2727, $\chi^2$=363.29, P<0.001). Meanwhile, their symptom onset dates were also earlier than other employees' in the market (**Figure S2**). The infections demonstrated spatial clusters in the basement, and highly clustered cases were identified in the seafood section (**Table S1**, **Figure 1A** and **S3**).

We further identified 14 booths (**Figure 1A** and **S4**) in XFDM-TH with both employee infections and environmental contaminations, and 3,294 individuals who visited these booths from May 20 to May 31. Serological screenings identified five visitors positive for IgG/IgM antibodies against SARS-CoV-2, and they had all been to the booth #S14. In contrast, no other booth was visited by more than two of these five visitors. All five visitors were negative for qRT-PCR, and none of their close contacts was infected based on qRT-PCR and antibody tests. These five individuals visited booth #S14 on May 30 or 31, and had not been to XFDM thereafter, suggesting that the virus was introduced into XFDM before June, which corresponds well to the putative starting time of this outbreak. All 7 employees of booth #S14 were infected evidenced by qRT-PCR and antibody detections, which further supported the possibility of booth #S14 being the source of acquisition (**Figure 1B**). Booth #S14 employees were also among the ones with early symptom onset time (**Figure 1C**).

To further investigate the origin of this outbreak, we sequenced 110 samples [5-7] and obtained 72 high-quality SARS-CoV-2 genome sequences (**Figure S5**). Notably, all genome sequences shared eight mutations (**Figure 1D**), with 38 sequences carrying only these eight mutations. The most common additional mutation, C12085T, was only shared by seven samples (**Figure S6**), suggesting that one ancestral virus strain (XFDM strain) was introduced into this outbreak. This XFDM strain sequence

ORIGINAL UNEDITED MANUSCRIPT

is obviously different from the viruses in two preceding outbreaks in China (Harbin and Shulan) and the sequences obtained in March, 2020 in Beijing (**Figure 1D**), indicating that the XFDM strain was unlikely to be derived from strains previously circulating in China. Phylogenetic analysis by Pangolin COVID-19 Lineage Assigner assigned the XFDM strain to clade B.1.1 [8] (**Figure 1D**). The ancestral sequences with seven mutations (without C6026T) were mainly identified in Europe (86.0%) (**Figure S7**). Thus, we speculated that XFDM strain was likely to be an imported strain.

To exclude other origins of infection, we conducted thorough epidemiological investigations on each infected individual. No employees of booth #S14 or their close contacts had been to medium/high-risk areas of Covid-19 epidemic or had contact with people from these areas. Thus, the SARS-CoV-2 in XFDM was possibly introduced through environmental routes. Salmon was the only imported commodity sold at booth #S14. We examined all salmon in the original sealed package in the cold storage which located outside XFDM, and six out of 3582 samples were positive for SARS-CoV-2 RNA. Notably, five positive fish were from company X, which supplied the salmon to booth #S14 on May 30. Through genome sequencing we obtained a significant number of SARS-CoV-2 reads from one swab of company X salmon. A total of 16,341 nucleotides on the viral genome, including three of the eight mutated positions, were covered by at least one read (**Figure 1E**). The genotypes of these three positions were identical to the XFDM strain. The possibility that the virus in the fish swab shared at least seven mutations with the XFDM strain was 60% by imputation analysis.

Given the abovementioned facts, we speculate that the Covid-19 resurgence in Beijing was likely to be initiated by an environment-to-human transmission originated

ORIGINAL UNEDITED MANUSCRIPT

from contaminated imported food via cold-chain logistics. Notably, a recent study found that SARS-CoV-2 showed no decline in infectivity after 21 days at 4 ℃ and -20 ℃ on the surface of chicken, salmon, and pork pieces [9], indicating that the survival period and transmission distance of the virus could be prolonged by cold-chain transportation of contaminated food.

Although it is unclear whether the viral load on the salmon is sufficient to establish an infection, the risk from the food and environment contamination exists [9,10]. Supply of the contaminated salmon and the exposure of early patients to booth #S14 both happened on May 30th, suggesting that co-exposure drove the very early stage of infection. Our finding is particularly important for countries where community transmissions are contained or suppressed. The virus could be reintroduced via cold-chain transportation of contaminated items and might initiate an outbreak. Even with low probability, such viral transmission would cause large scale outbreaks if not being intervened immediately after the first cases. Regional guidelines on Covid-19 prevention and control should integrate surveillance of the cold-chain imported products, especially those from epidemic regions for the Covid-19.

**SUPPLEMENTARY DATA**

Supplementary data are available at NSR online.

**ACKNOWLEDGEMENTS**

We thank the health workers who contributed to the epidemiological survey, sample collection and transportation. We thank Ms. Chenyang Geng, Ms. Jing Sun, Beijing Advanced Innovation Center for Genomics (ICG) and Biomedical Pioneering

ORIGINAL UNEDITED MANUSCRIPT

Innovation Center (BIOPIC) at Peking University for the support on high throughput sequencing.

**FUNDING**

This study is funded by The National Major Science & Technology Project for Control and Prevention of Major Infectious Diseases in China (2017ZX10103004), European Union Horizon 2020 (MOOD 874850), Beijing Advanced Innovation Center for Structural Biology (ICSB), and Beijing Advanced Innovation Center for Genomics (ICG).

***Conflict of interest statement*.** None declared.

**REFERENCES**

1. World Health Organization. WHO Coronavirus Disease (COVID-19) Dashboard. https://covid19.who.int/ (accessed August 29, 2020).

2. Ren LL, Wang YM, Wu ZQ, et al. Identification of a novel coronavirus causing severe pneumonia in human: a descriptive study. *Chin Med J (Engl)* 2020;**133**:1015-24.

3. Ceylan Z, Meral R, Cetinkaya T. Relevance of SARS-CoV-2 in food safety and food hygiene: potential preventive measures, suggestions and nanotechnological approaches. *Virusdisease* 2020; **31**:154-60.

4. European Commission. Food Safety and the Coronavirus Disease 2019 (COVID-19). https://www.fda.gov/food/food-safety-during-emergencies/food-safety-and-coronavirus-disease-2019-covid-19 (accessed Aug 16, 2020).

5. Di L, Fu Y, Sun Y, et al. RNA sequencing by direct tagmentation of RNA/DNA hybrids. *Proc Natl Acad Sci U S A* 2020; **117**:2886-93.

6. Chen C, Li J, Di L, et al. MINERVA: a facile strategy for SARS-CoV-2 whole genome deep sequencing of clinical samples. *bioRxiv* 2020.

ORIGINAL UNEDITED MANUSCRIPT

https://www.biorxiv.org/content/10.1101/2020.04.25.060947v2

7. Xu Y, Kang L, Shen Z, et al. Hybrid capture-based sequencing enables unbiased recovery of SAR-CoV-2 genomes from fecal samples and characterization of the dynamics of intra-host variants. *bioRxiv* 2020. https://www.biorxiv.org/content/10.1101/2020.07.30.230102v1

8. Rambaut A, Holmes E, O'Toole A, et al. A dynamic nomenclature proposal for SARS-CoV-2 lineages to assist genomic epidemiology. *Nat Microbial* 2020. https://doi.org/10.1038/s41564-020-0770-5.

9. Fisher D, Reilly A, Zheng A, Cook A, Anderson D. Seeding of outbreaks of COVID-19 by contaminated fresh and frozen food. *BioRxiv* 2020. https://doi.org/10.1101/2020.08.17.255166.

10. Hoseinzadeh E, Safoura Javan, Farzadkia M, Mohammadi F, Hossini H, Taghavi M. An updated min-review on environmental route of the SARS-CoV-2 transmission. *Ecotoxicol Environ Saf* 2020; 202:111015.

ORIGINAL UNEDITED MANUSCRIPT

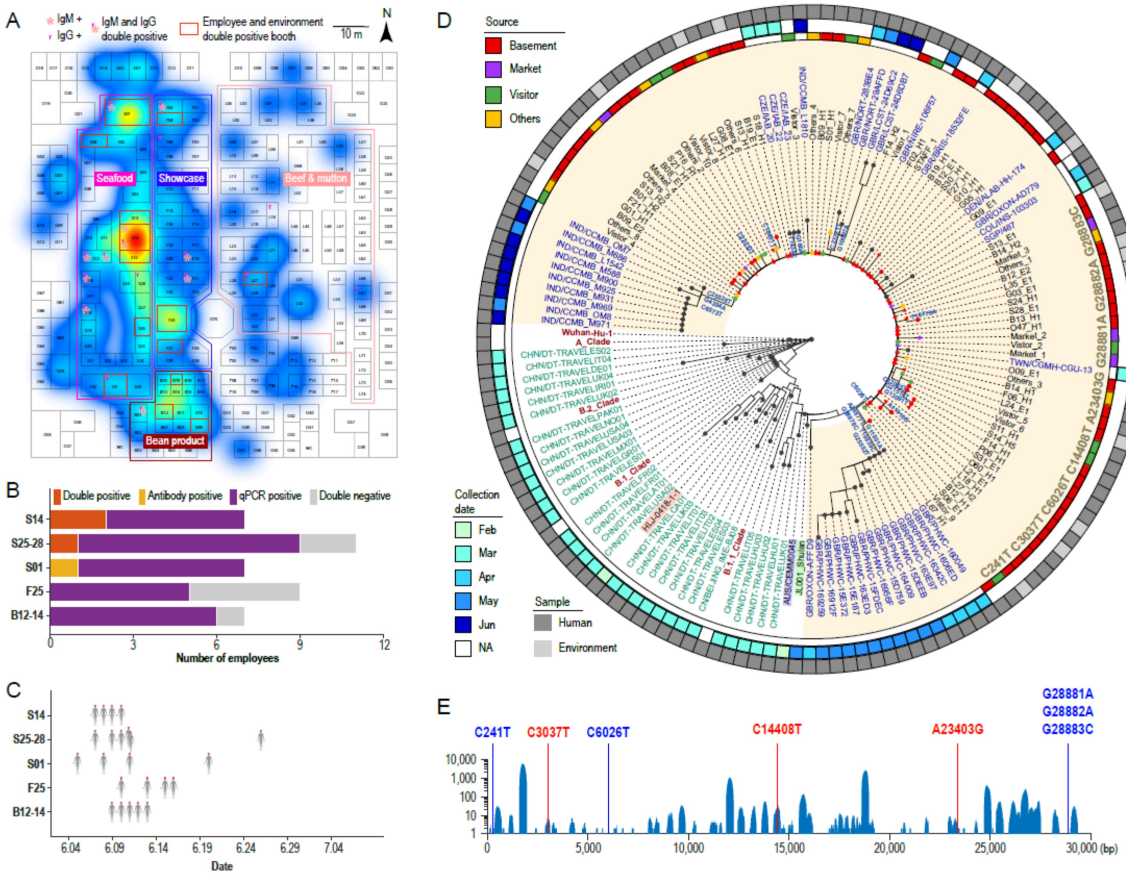

**Figure 1. Identification of the possible source of infection and analysis of viral genomes obtained in the outbreak.** (A) Heatmap showing the density distribution of infected employees in the basement of XFDM-TH reveals several possible originating sites. Employee and environment double-positive booths are highlighted in red frame. Cases positive for IgM/IgG antibodies against SARS-CoV-2 are marked in orange and pink, respectively. The numbers of booths are as follows, seafood:32, showcase:29, bean product:20, beef and mutton:74. (B) SARS-CoV-2 RNA and antibody test results of employees in suspected booths. Booth #S14 employees had the highest infection rates (100%) determined by qRT-PCR and antibody detections. (C) Symptom onset dates of employees in suspected booths. Booth #S14 employees had relatively early symptom onset time. (D) Phylogenetic tree of high-quality genomes obtained in the

ORIGINAL UNEDITED

outbreak. From inside to outside: tree structure, sequence IDs, sample source, collection date, and sample type. The clade brunch that includes all strains with the same eight mutations with XFDM strain is highlighted in yellow background. The IDs of sequences obtained in the cases before this outbreak in Beijing are marked in green. The sequence IDs of two preceding outbreaks in China (Harbin HLJ-0418 and Shulan JL001_Shulan) are highlighted in red and green backgrounds, respectively. IDs of all SARS-CoV-2 sequences in 2019nCoVR (until 12 September 2020) that share the same eight mutations with XFDM strain are marked in blue. The IDs of sequences with only seven mutations (except C6026T) in 2019nCoVR (AUS/CEMM0045) are highlighted in blue background. Mutations that shared by at least two sequences are labelled on the tree. Reference genome (Wuhan-hu-1) and four main clades (A, B.1, B.2, B.1.1) are also included in the phylogenetic tree. The XFDM sample ID was started with the booth number and followed by either H (employee) or E (environment) and a number starting from 1. (E) Genome coverage of the SARS-CoV-2 recovered from the salmon swab. All identified mutations are labelled in red, which are also observed in the XFDM strain. Another five mutations in the XFDM strain are not identified due to the lack of sequence coverage in these regions (labelled in blue).

ORIGINAL UNEDITED MANUSCRIPT
