# Cold-chain food contamination as the possible origin of COVID-19 resurgence in Beijing

**DOI:** 10.1093/nsr/nwaa264

**Published:** 2020-10-23

**Authors:** Xinghuo Pang, Lili Ren, Shuangsheng Wu, Wentai Ma, Jian Yang, Lin Di, Jie Li, Yan Xiao, Lu Kang, Shichang Du, Jing Du, Jing Wang, Gang Li, Shuguang Zhai, Lijuan Chen, Wenxiong Zhou, Shengjie Lai, Lei Gao, Yang Pan, Quanyi Wang, Mingkun Li, Jianbin Wang, Yanyi Huang, Jianwei Wang, COVID-19 Field Response Group, COVID-19 Laboratory Testing Group

**Affiliations:** Beijing Center for Disease Prevention and Control (CDC), China; Research Centre for Preventive Medicine of Beijing, China; NHC Key Laboratory of Systems Biology of Pathogens and Christophe Mérieux Laboratory, Institute of Pathogen Biology, Chinese Academy of Medical Sciences & Peking Union Medical College, China; Key Laboratory of Respiratory Disease Pathogenomics, Chinese Academy of Medical Sciences and Peking Union Medical College, China; Beijing Center for Disease Prevention and Control (CDC), China; Research Centre for Preventive Medicine of Beijing, China; Beijing Institute of Genomics, Chinese Academy of Sciences, and China National Center for Bioinformation, China; University of Chinese Academy of Sciences, China; NHC Key Laboratory of Systems Biology of Pathogens, Institute of Pathogen Biology, Chinese Academy of Medical Sciences & Peking Union Medical College, China; Beijing Advanced Innovation Center for Genomics (ICG), Biomedical Pioneering Innovation Center (BIOPIC), College of Chemistry, and Peking-Tsinghua Center for Life Sciences, Peking University, China; School of Life Sciences, Tsinghua-Peking Center for Life Sciences, Tsinghua University, China; NHC Key Laboratory of Systems Biology of Pathogens and Christophe Mérieux Laboratory, Institute of Pathogen Biology, Chinese Academy of Medical Sciences & Peking Union Medical College, China; Key Laboratory of Respiratory Disease Pathogenomics, Chinese Academy of Medical Sciences and Peking Union Medical College, China; Beijing Institute of Genomics, Chinese Academy of Sciences, and China National Center for Bioinformation, China; University of Chinese Academy of Sciences, China; Beijing Center for Disease Prevention and Control (CDC), China; Research Centre for Preventive Medicine of Beijing, China; Beijing Center for Disease Prevention and Control (CDC), China; Research Centre for Preventive Medicine of Beijing, China; Beijing Center for Disease Prevention and Control (CDC), China; Research Centre for Preventive Medicine of Beijing, China; Beijing Center for Disease Prevention and Control (CDC), China; Research Centre for Preventive Medicine of Beijing, China; Beijing Center for Disease Prevention and Control (CDC), China; Research Centre for Preventive Medicine of Beijing, China; Beijing Center for Disease Prevention and Control (CDC), China; Research Centre for Preventive Medicine of Beijing, China; Beijing Advanced Innovation Center for Genomics (ICG), Biomedical Pioneering Innovation Center (BIOPIC), College of Chemistry, and Peking-Tsinghua Center for Life Sciences, Peking University, China; WorldPop, School of Geography and Environmental Science, University of Southampton, UK; NHC Key Laboratory of Systems Biology of Pathogens, Institute of Pathogen Biology, Chinese Academy of Medical Sciences & Peking Union Medical College, China; Beijing Center for Disease Prevention and Control (CDC), China; Research Centre for Preventive Medicine of Beijing, China; Beijing Center for Disease Prevention and Control (CDC), China; Research Centre for Preventive Medicine of Beijing, China; Beijing Institute of Genomics, Chinese Academy of Sciences, and China National Center for Bioinformation, China; University of Chinese Academy of Sciences, China; Center for Excellence in Animal Evolution and Genetics, Chinese Academy of Sciences, China; School of Life Sciences, Tsinghua-Peking Center for Life Sciences, Tsinghua University, China; Beijing Advanced Innovation Center for Structural Biology (ICSB), Tsinghua University, China; Chinese Institute for Brain Research (CIBR), China; Beijing Advanced Innovation Center for Genomics (ICG), Biomedical Pioneering Innovation Center (BIOPIC), College of Chemistry, and Peking-Tsinghua Center for Life Sciences, Peking University, China; Institute for Cell Analysis, Shenzhen Bay Laboratory, China; NHC Key Laboratory of Systems Biology of Pathogens and Christophe Mérieux Laboratory, Institute of Pathogen Biology, Chinese Academy of Medical Sciences & Peking Union Medical College, China; Key Laboratory of Respiratory Disease Pathogenomics, Chinese Academy of Medical Sciences and Peking Union Medical College, China; Beijing Center for Disease Prevention and Control (CDC), China; Research Centre for Preventive Medicine of Beijing, China; Beijing Center for Disease Prevention and Control (CDC), China; Research Centre for Preventive Medicine of Beijing, China

COVID-19, caused by SARS-CoV-2 [1,2], has been contained in China through stringent non-pharmaceutical interventions. Border control and quarantine have effectively prevented the virus from being spread by infected travellers, but the risk of resurgence caused by other routes of introduction and transmission remains unclear, and current strategies to prevent resurgence could be flawed. Since July, SARS-CoV-2 RNA contaminations in frozen food imported from countries with ongoing epidemics have been reported in nine provinces in China [3,4]. However, there is no robust evidence of COVID-19 outbreaks initiated by environment-to-human transmission. Here we add to evidence of such transmission by investigating the recent COVID-19 resurgence in Beijing.

On 11 June 2020, a 52-year old man suffering from fever and cough was diagnosed with COVID-19 in Beijing, after a 56-day zero new case interval. He had no exposure history of known COVID-19 cases. On 12 June, 112 close contacts of the index case and 242 environmental samples collected from the places that he had visited were tested by quantitative reverse transcription polymerase chain reaction (qRT-PCR).

 All close contacts were negative, but two environmental samples from Xinfadi Market (XFDM) were positive for SARS-CoV-2. This led to in-depth investigation to confirm the role of XFDM in virus spread. A total of 538 employees from the booths that were close to the SARS-CoV-2-positive environmental samples were tested, and 45 were positive by qRT-PCR.

To evaluate the extent of infection spreading, a screening campaign of SARS-CoV-2 infection was implemented over the city by Beijing Center for Disease Prevention and Control. Between 15 June and 10 July, a total of more than 10 million citizens, and 5342 environmental samples were screened. Eventually 368 qRT-PCR positive cases were confirmed (Fig. S1A), of which 169 (45.9%) had a history of working in XFDM. Of the visitors to XFDM between May 30 and 12 June, 103 (28.0%) were diagnosed. The remaining 96 (26.1%) patients had contact with the infected employees or visitors. These findings suggested a single outbreak source in Beijing (Fig. S1B). Retrospective epidemiological investigation revealed the earliest symptom onset of a patient on 4 June (Fig. S1C).

To probe the origin of the infection, we analysed the spatial distribution of infected employees in XFDM. Strikingly, 20.9% (122/584) of employees working in the basement of the XFDM trading hall (XFDM-TH) were positive for SARS-CoV-2, which is significantly higher than those of other areas in the market (1.7%, 47/2727, χ^2 ^= 363.29, *P* < 0.001). Meanwhile, their symptom onset dates were also earlier than other employees in the market (Fig. S2). The infections demonstrated spatial clusters in the basement, and highly clustered cases were identified in the seafood section (Table S1, Figs 1A and S3).

**Figure 1.**
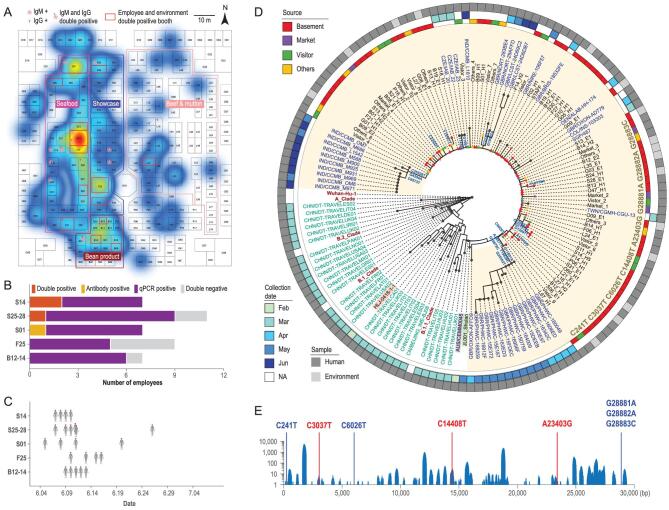
Identification of the possible source of infection and analysis of viral genomes obtained in the outbreak. (A) Heatmap showing the density distribution of infected employees in the basement of XFDM-TH reveals several possible originating sites. Employee and environment double-positive booths are highlighted in a red frame. Cases positive for IgM/IgG antibodies against SARS-CoV-2 are marked in orange and pink, respectively. The numbers of booths are as follows, seafood: 32, showcase: 29, bean product: 20, beef and mutton: 74. (B) SARS-CoV-2 RNA and antibody test results of employees in suspected booths. Booth #S14 employees had the highest infection rates (100%) determined by qRT-PCR and antibody detections. (C) Symptom onset dates of employees in suspected booths. Booth #S14 employees had relatively early symptom onset time. (D) Phylogenetic tree of high-quality genomes obtained in the outbreak. From inside to outside: tree structure, sequence IDs, sample source, collection date and sample type. The clade brunch that includes all strains with the same eight mutations as the XFDM strain is highlighted with a yellow background. The IDs of sequences obtained in the cases before this outbreak in Beijing are marked in green. The sequence IDs of two preceding outbreaks in China (Harbin HLJ-0418 and Shulan JL001_Shulan) are highlighted in red and green backgrounds, respectively. IDs of all SARS-CoV-2 sequences in 2019nCoVR (until 12 September 2020) that share the same eight mutations with the XFDM strain are marked in blue. The IDs of sequences with only seven mutations (except C6026T) in 2019nCoVR (AUS/CEMM0045) are highlighted with a blue background. Mutations shared by at least two sequences are labelled on the tree. Reference genome (Wuhan-hu-1) and four main clades (A, B.1, B.2, B.1.1) are also included in the phylogenetic tree. The XFDM sample ID was started with the booth number and followed by either H (employee) or E (environment) and a number starting from 1. (E) Genome coverage of the SARS-CoV-2 recovered from the salmon swab. All identified mutations are labelled in red, which are also observed in the XFDM strain. Another five mutations in the XFDM strain are not identified due to the lack of sequence coverage in these regions (labelled in blue).

We further identified 14 booths (Figs 1A and S4) in XFDM-TH with both employee infections and environmental contaminations, and 3294 individuals who visited these booths from 20 to 31 May. Serological screenings identified five visitors positive for IgG/IgM antibodies against SARS-CoV-2, and they had all been to the booth #S14. In contrast, no other booth was visited by more than two of these five visitors. All five visitors were negative for qRT-PCR, and none of their close contacts was infected based on qRT-PCR and antibody tests. These

five individuals visited booth #S14 on 30 or 31 May, and had not been to XFDM thereafter, suggesting that the virus was introduced into XFDM before June, which corresponds well to the putative starting time of this outbreak. All 7 employees of booth #S14

were infected evidenced by qRT-PCR and antibody detections, which further supported the possibility of booth #S14 being the source of acquisition (Fig. 1B). Booth #S14 employees were also among the ones with early symptom onset time (Fig. 1C).

To further investigate the origin of this outbreak, we sequenced 110 samples [5–7] and obtained 72 high-quality SARS-CoV-2 genome sequences (Fig. S5). Notably, all genome sequences shared eight mutations (Fig. 1D), with 38 sequences carrying only

these eight mutations. The most common additional mutation, C12085T, was only shared by seven samples (Fig. S6), suggesting that one ancestral virus strain (XFDM strain) was introduced into this outbreak. This XFDM strain sequence is obviously different from the viruses in two preceding outbreaks in China (Harbin and Shulan) and the sequences obtained in March 2020 in Beijing (Fig. 1D), indicating that the XFDM strain was unlikely to be derived from strains previously circulating in China. Phylogenetic analysis by Pangolin COVID-19 Lineage Assigner assigned the XFDM strain to clade B.1.1 [8] (Fig. 1D). The ancestral sequences with seven mutations (without C6026T) were mainly identified in Europe (86.0%) (Fig. S7). Thus, we speculated that the XFDM strain was likely to be an imported strain.

To exclude other origins of infection, we conducted thorough epidemiological investigations on each infected individual. No employees of booth #S14 or their close contacts had been to medium/high-risk areas of the COVID-19 epidemic or had contact with people from these areas. Thus, the SARS-CoV-2 in XFDM was possibly introduced through environmental routes. Salmon was the only imported commodity sold at booth #S14. We examined all salmon in the original sealed package in the cold storage which was located outside XFDM, and six out of 3582 samples were positive for SARS-CoV-2 RNA. Notably, five positive fish were from company X, which supplied the salmon to booth #S14 on May 30. Through genome sequencing we obtained a significant number of SARS-CoV-2 reads from one swab of company X salmon. A total of 16 341 nucleotides on the viral genome, including three of the eight mutated positions, were covered by at least one read (Fig. 1E). The genotypes of these three positions were identical to the XFDM strain. The possibility that the virus in the fish swab shared at least seven mutations with the XFDM strain was 60% by imputation analysis.

Given the abovementioned facts, we speculate that the COVID-19 resurgence in Beijing was likely to be initiated by an environment-to-human transmission originated from contaminated imported food via cold-chain logistics. Notably, a recent study found that SARS-CoV-2 showed no decline in infectivity after 21 days at 4°C and −20°C on the surface of chicken, salmon, and pork pieces [9], indicating that the survival period and transmission distance of the virus could be prolonged by cold-chain transportation of contaminated food.

Although it is unclear whether the viral load on the salmon is sufficient to establish an infection, the risk from the food and environment contamination exists [9,10]. Supply of the contaminated salmon and the exposure of early patients to booth #S14 both happened on May 30, suggesting that co-exposure drove the very early stage of infection. Our finding is particularly important for countries where community transmissions are contained or suppressed. The virus could be reintroduced via cold-chain transportation of contaminated items and might initiate an outbreak. Even with low probability, such viral transmission would cause large scale outbreaks if not being intervened immediately after the first cases. Regional guidelines on COVID-19 prevention and control should integrate surveillance of cold-chain imported products, especially those from epidemic regions of COVID-19.

## Supplementary Material

nwaa264_Supplemental_File
