# Supplementary material for: Cold-chain food contamination as the possible origin of COVID-19 resurgence in Beijing
Source: Natl Sci Rev. 2020 Oct 23;7(12):1861–4. doi: 10.1093/nsr/nwaa264 (PMC7665658; doi:10.1093/nsr/nwaa264)
Supplement: nwaa264_Supplemental_File [file nwaa264_supplemental_file.docx]

**Supplementary materials**

**Cold-chain food contamination as the possible origin of Covid-19 resurgence in Beijing**

Xinghuo Pang^1,2,†^, Lili Ren^3,4,†^, Shuangsheng Wu^1,2,†^, Wentai Ma^5,6,†^, JianYang^7^, Lin Di^8^, Jie Li^9^, Yan Xiao^3,4^, Lu Kang^5,6^, Shichang Du^1,2^, Jing Du,^1,2^ Jing Wang^1,2^, Gang Li^1,2^, Shuguang Zhai^1,2^, Lijuan Chen^1,2^, Wenxiong Zhou^8^, Shengjie Lai^10^, Lei Gao^7^, Yang Pan^1,2,^*, Quanyi Wang^1,2,^*, Mingkun Li^5,6,11,^*, Jianbin Wang^9,12,13,^*, Yanyi Huang^8,14,^*, Jianwei Wang^3,4,^*, COVID-19 Field Response Group^1,2^ , and COVID-19 Laboratory Testing Group^1,2^

^1^ Beijing Center for Disease Prevention and Control (CDC), Beijing 100013, China

^2^ Research Centre for Preventive Medicine of Beijing, Beijing 100013, China

^3^ NHC Key Laboratory of Systems Biology of Pathogens and Christophe Mérieux Laboratory, Institute of Pathogen Biology, Chinese Academy of Medical Sciences & Peking Union Medical College, Beijing 100730, China

^4^ Key Laboratory of Respiratory Disease Pathogenomics, Chinese Academy of Medical Sciences and Peking Union Medical College, Beijing 100730, China

^5^ Beijing Institute of Genomics, Chinese Academy of Sciences, and China National Center for Bioinformation, Beijing 100101, China

^6^ University of Chinese Academy of Sciences, Beijing 100049, China

^7^ NHC Key Laboratory of Systems Biology of Pathogens, Institute of Pathogen Biology, Chinese Academy of Medical Sciences & Peking Union Medical College, Beijing 100730, China

^8^ Beijing Advanced Innovation Center for Genomics (ICG), Biomedical Pioneering Innovation Center (BIOPIC), College of Chemistry, and Peking-Tsinghua Center for Life Sciences, Peking University, Beijing 100871, China

^9^ School of Life Scences, Tsinghua-Peking Center for Life Sciences, Tsinghua University, Beijing 100084, China

^10^ WorldPop, School of Geography and Environmental Science, University of Southampton, England SO17 1BJ, UK

^11^ Center for Excellence in Animal Evolution and Genetics, Chinese Academy of Sciences, Kunming 650223, China

^12^ Beijing Advanced Innovation Center for Structural Biology (ICSB), Tsinghua University, Beijing 100084, China.

^13^ Chinese Institute for Brain Research (CIBR), Beijing 102206, China

^14^ Institute for Cell Analysis, Shenzhen Bay Laboratory, Guangdong 518132, China

^†^ Equally contributed to this work.

* Corresponding authors. E-mails: wangjw28@163.com (J. W.), yanyi@pku.edu.cn (Y.H.), jianbinwang@tsinghua.edu.cn (Jianbin W.), limk@big.ac.cn (M. L.), pan_yang@126.com (Y. P.), bjcdcxm@126.com (Q. W.)

**Supporting Methods**

**Epidemiological investigation.** The epidemiological investigations of Covid-19 patients, suspected individuals, and their close contacts were conducted within 24 hours upon receiving the report. The information registered includes demographic data, time of symptom onset, travel history and itinerary, close contacts history, and exposure history to XFDM. To identify all visitors to the XFDM during the outbreak and three weeks before the outbreak, especially those visited contaminated booths, records from epidemiological investigations were retrieved and analyzed.

**Sample collection.** Oropharyngeal swabs from more than 10 million citizens, 9,492 individuals suspected to be infected with SARS-CoV-2 and all 3,311 employees working at XFDM were collected and stored in viral transportation medium. Peripheral venous blood was collected for antibody detections. A thorough survey of all business sites in XFDM was carried out, and 5,342 environmental samples were collected from the surface in each vending booth (cutting board, utensils, processing bench, washbasin, faucet, sewer, and trash bin), public area (elevator button, stair handrail, door handle, trash bin, drain, sewer, faucets, urinals, and toilet), as well as food, packages, and cold storages. Body swabs and package swabs from the salmon fish sealed in the original packages in the supplier’s cold storage outside XFDM were also collected. The data collection for cases was deemed by the National Health Commission as the contents of public health outbreak investigation.

**SARS-CoV-2 RNA detection.** Total nucleic acids were extracted using an automated preparation system (Thermo Fisher Scientific, Carlsbad, CA, USA). qRT-PCR was performed according to the protocol recommended by China National Health Commission [1].

**Metatranscriptomic sequencing library construction.** The extracted nucleic acids were reverse transcribed with random primers, and sequencing libraries were constructed using an RNA/DNA hybrid tagmentation pipeline [2,3] or NuGEN Trio RNA-Seq (NuGEN, San Carlos, CA) following the manufacturer’s protocol. The library was enriched by using targeted SARS-CoV-2 gene probes (iGeneTech, Beijing, China) [4]. Libraries were sequenced on an Illumina NextSeq 500 platform with 2×75 paired-end mode. Negative control samples were processed and sequenced in parallel for each sequencing run as contamination control.

**SARS-CoV-2 genome sequence analysis.** Quality control and adaptor trimming was done by FASTP (-l 50 --cut_tail –cut_tail_mean_quality 20) [5]. Taxonomic classification was assigned by kraken2 [6]. The clean reads that assigned to Cornidovirineae were mapped to the SARS-CoV-2 genome (GenBank: MN908947.3) using the BWA-MEM software (a minimum of 50 matches were required) [7]. Deduplication was performed with Picard toolkit (1.119) [8]. The nucleotide mpileup file and the read counts file were generated with SAMtools and Varscan2 [9,10], respectively. A core set of mutations was identified using stringent criteria, 1) Coverage ≥ 5 folds; 2) Strand bias (calculated by VarScan2) was between 0·1 and 0·9; 3) Frequency of the mutation ≥ 70%; 4) the mutation was supported by internal of the reads (10 bp on both ends were removed). Genetic variations of the virus from each individual were identified based on a loser criterion that requires the frequency of mutation ≥ 50%. Only mutations included in the core set were considered. Phylogenetic analysis was conducted using MEGA X [11], and the evolutionary distances were computed using the Maximum Composite Likelihood method, ambiguous positions were removed for each sequence pair (pairwise deletion). The viral genomes reported in this study have been deposited in the Genome Warehouse in the National Genomics Data Center (under project PRJCA002866, publicly accessible at https://bigd.big.ac.cn/gsa). For comparison, SARS-CoV-2 variants were retrieved from 2019nCoVR (September 12, 2020), which included data from 52 841 high quality viral genomes.

**Antibody detections.** The immunoglobin (Ig) M and IgG against SARS-CoV-2 were detected by using chemiluminescence method according to the manufacture’s protocol (Sinbe, Shenzhen, China) in all employees and visitors of 14 critical booths, which were identified if both employees and related environment samples showed positive for viral RNA.

**Data analysis**. The start date of the outbreak was determined according to the date of illness onset of cases obtained from epidemiological investigations. Then all the visitors and employees related to XFDM two weeks before the deduced start time were involved for further screening. The earlier symptom onset date, higher positive rate of SARS-CoV-2-RNA and viral antibody in employee, higher viral RNA detection rate in environment were used to trace the potential source of acquisition. As all local supplies were from geographical regions with no reported cases, the imported goods and cold storage of the supplier of the deduced source booth were then tested. The genomic mutations were analyzed to decide the shared ancestor. To clarify the source of virus acquisition, we mapped the positive detections of SARS-CoV-2 RNA within the market. The number, positive rate, and locations of viral RNA-positive samples in employees and environment in each section and booth of XFDM were presented by kernel density heatmap. The significance of detection rates difference was calculated by Chi-square test. The correlation between the viral load (cycle threshold value) and genome coverage was analyzed by the Pearson correlations test. To estimate the probability that the incomplete genome recovered from the fish swab was identical to the XFDM strain. We have searched against 52 841 high quality viral genomes available at 2019nCoVR database (https://bigd.big.ac.cn/ncov/, Sep 12, 2020) and found 8684 sequences were identical to the fish swab sequences (only 10824 positions with sequencing depth ≥ 2 fold and major allele frequency ≥ 70% in the fish swab genome were taken into account). Among these matched sequences, 5270 sequences possessed all XFDM mutations (C241T, C3037T, C14408T, A23403G, G28881A, G2882A, G28883C) except C6026T. Thus, we deduced that the probability that the virus in the fish swab had all seven mutations identical to the XFDM strain was 60% (5270/8684).

**Table S1.** **Detection rate of samples collected from employees and environmental samples in Xinfadi market (XFDM)**

| Section in XFDM | Employees | | | Environmental samples | | |
| --- | --- | --- | --- | --- | --- | --- |
|  | Tested number | Case number | Positive rate（%） | Tested number | Positive number | Positive rate（%） |
| Trading hall | 584 | 122 | 20.9 | 2467 | 135 | 5.5 |
| Seafood | 101 | 52 | 51.5 | 780 | 62 | 7.9 |
| Seasoning | 33 | 8 | 24.2 | 86 | 6 | 7.0 |
| Bean products | 50 | 14 | 28.0 | 230 | 35 | 15.2 |
| Showcase | 75 | 19 | 25.3 | 353 | 19 | 5.4 |
| Beef and mutton | 189 | 14 | 7.4 | 598 | 12 | 2.0 |
| Other sections | 136 | 15 | 11.0 | 420 | 1 | 0.2 |
| Other regions in market | 2727 | 47 | 1.7 | 2875 | 4 | 0.1 |
| Total number | 3311 | 169 | 5.1 | 5342 | 139 | 2.6 |


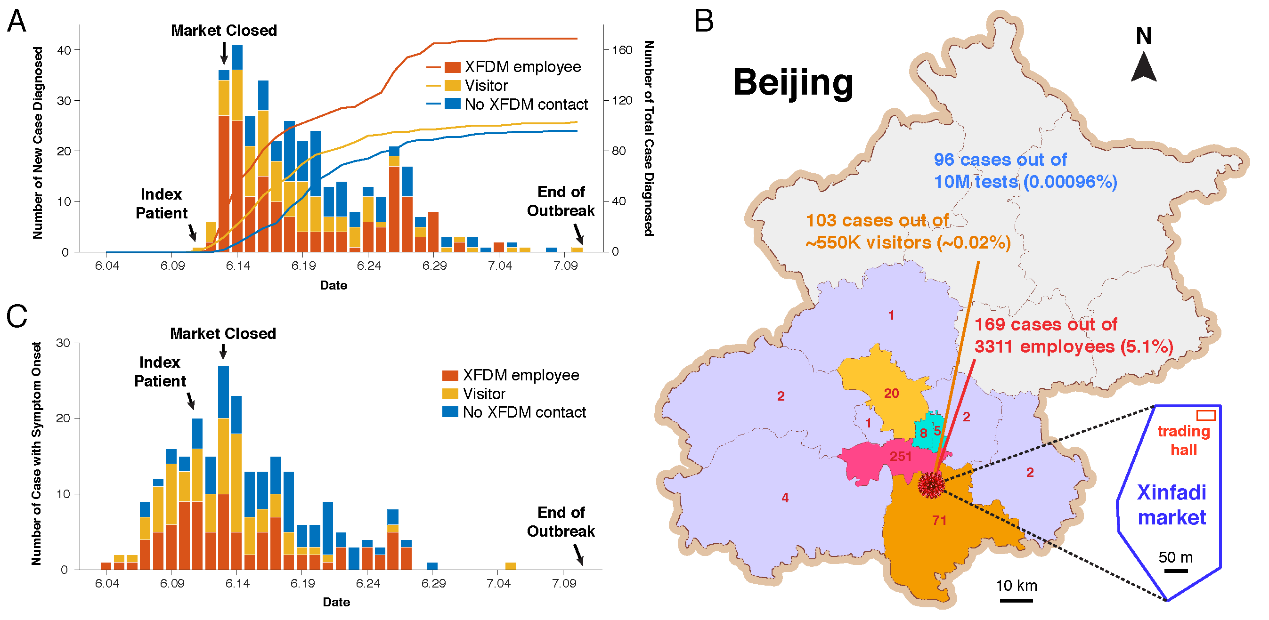
**Figure S1. Re-emergence of an outbreak of Covid-19 in Beijing between June and July 2020.** (A) Daily numbers of new cases reported. The curve line in red, blue and yellow colors represent the total number of confirmed cases in Xinfadi market (XFDM) employees, visitors to XFDM and cases have no history to the market. (B) Distribution of cases in Beijing and positive rates of SARS-CoV-2 detection in different categories. Xinfadi market is marked as a red dot on the map. (C) Daily numbers of cases with symptom onset.


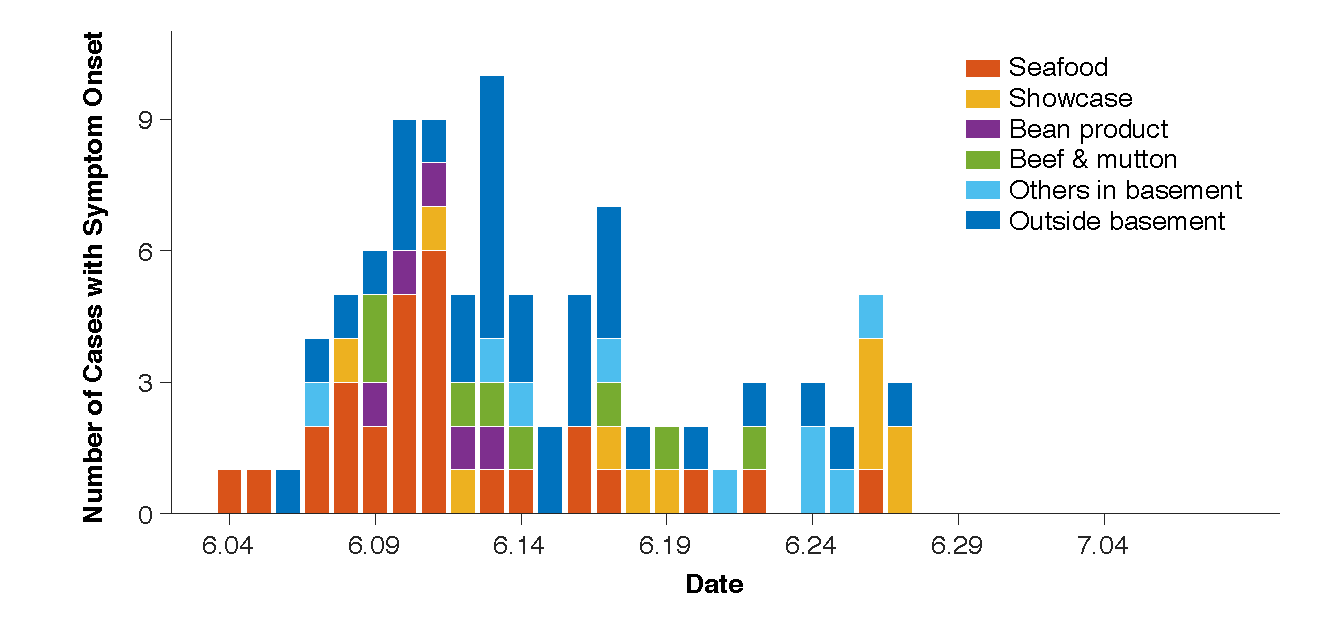


**Figure S2. Daily numbers of XFDM employee cases with symptom onset.** Employees working in different areas were labelled in different colors.


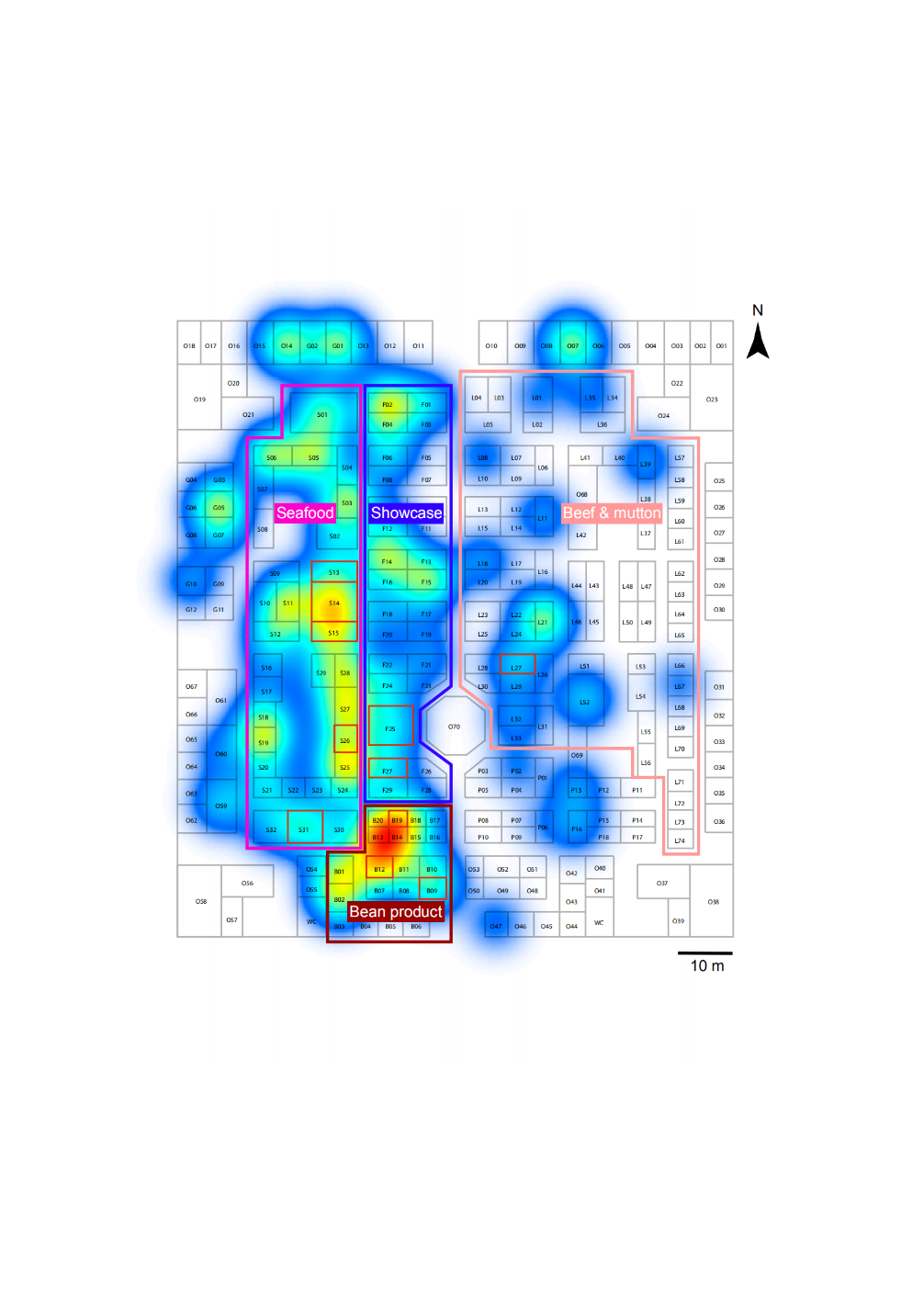


**Figure S3. Heatmap of employee detection rate in the basement of XFDM-TH reveals several possible originating sites.** Employee and environment double-positive booths are highlighted in red frame.


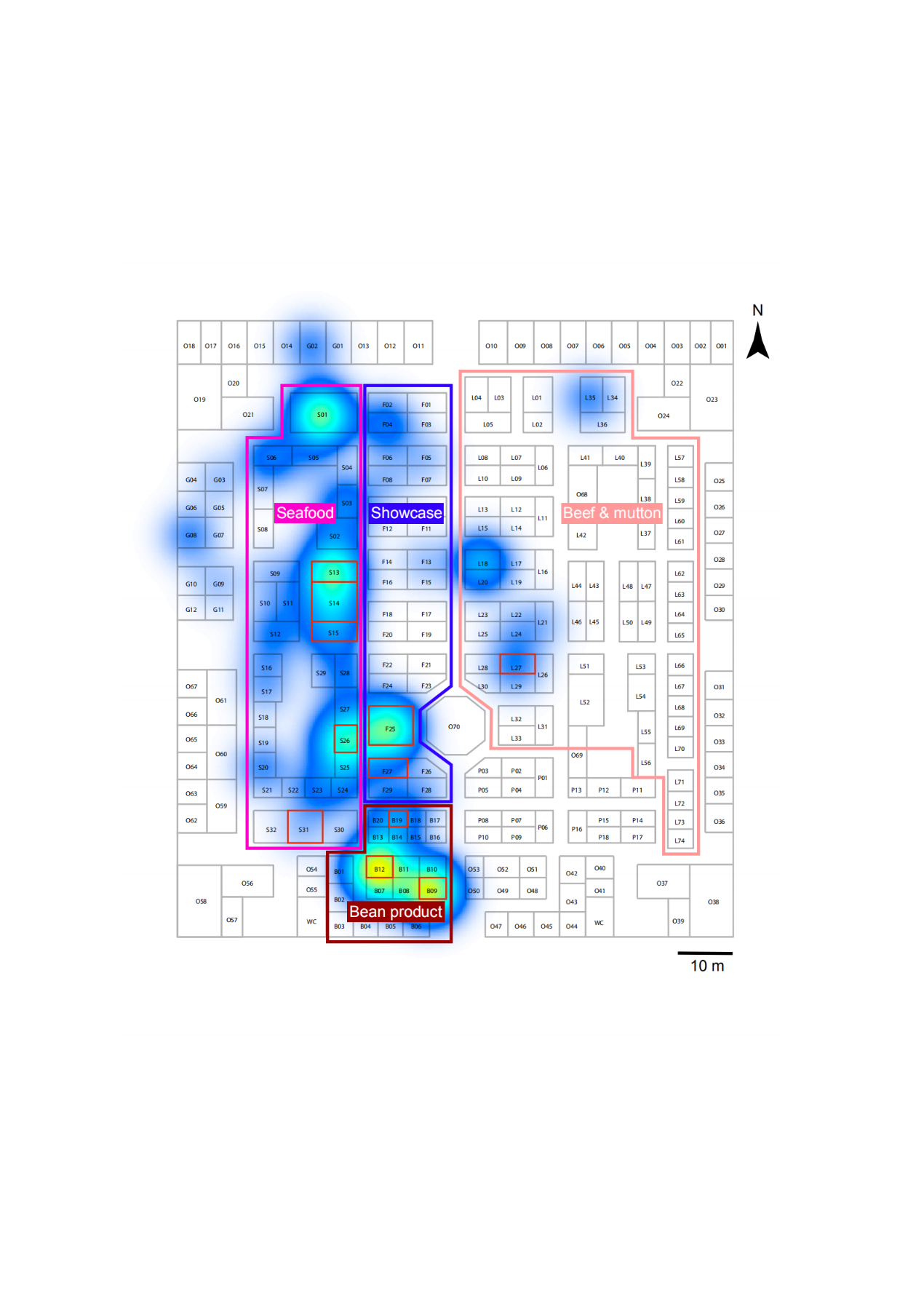
**Figure S4. Heatmap of positive environmental samples distribution in the basement of XFDM-TH reveals several possible originating sites.** Employee and environment double-positive booths are highlighted by red frame.


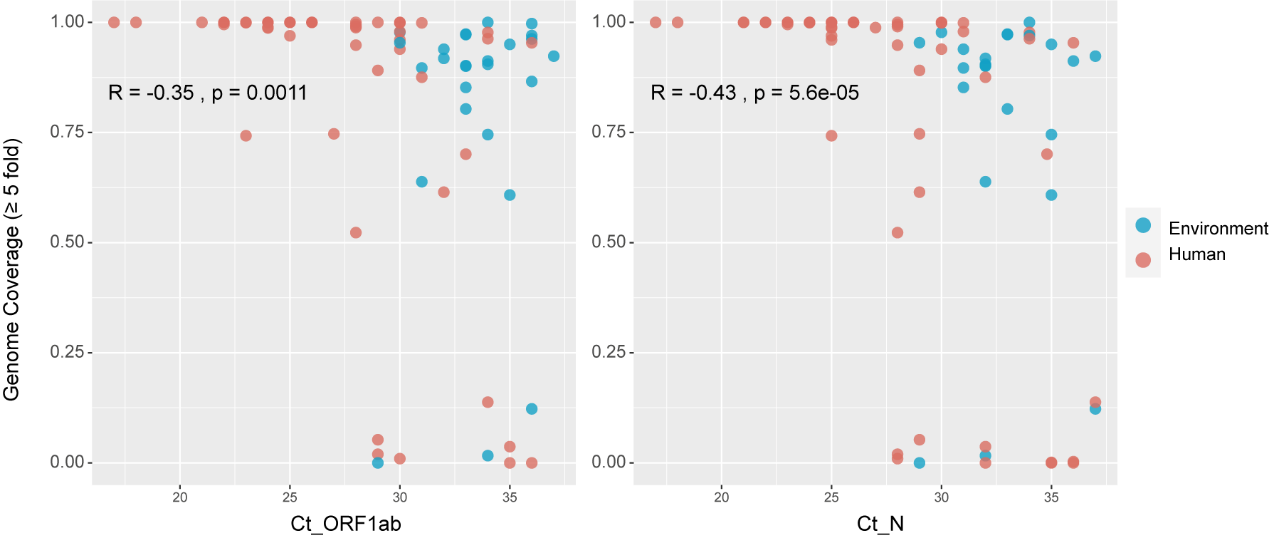


**Figure S5. Correlation between the proportion of recovered genome and cycle threshold (Ct) values.** Ct values were measured by probes targeting *ORF1ab* gene and *N* gene. Pearson correlation coefficients and *p*-values are labelled in the figure.

**
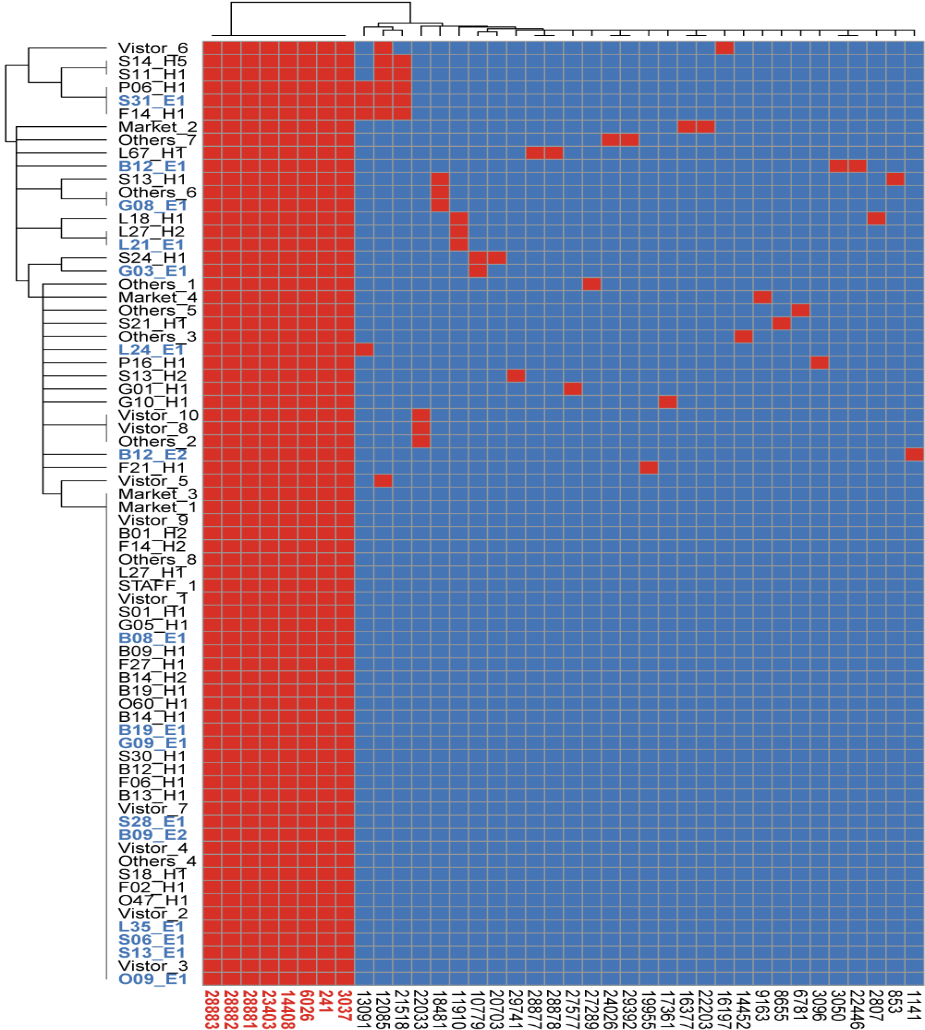
**

**Figure S6. Shareness of the mutations observed in samples from the XFDM outbreak.** Site mutations are labeled in red, wildtype alleles identical to the reference genome are labeled in blue. Sequence ID of human and environmental samples are labeled in black and blue, respectively.

**Figure S7. Distribution of SARS-CoV-2 strains in the world that share the same mutations with XFDM strain.** (A) Distribution of strains with only seven mutations (C241T, C3037T, C14408T, A23403G, G28881A, G28882A, G28883C). (B) Distribution of strains with eight mutations (C241T, C3037T, C6026T, C14408T, A23403G, G28881A, G28882A, G28883C). The number of sequences are labelled at the top of each histogram bar.

**REFERENCES**

1. Chinese Center for Disease Control and Prevention. Guidelines of laboratory testing for novel coronavirus in 2019. http://www.chinacdc.cn/jkzt/crb/zl/szkb_11803/jszl_11815/202003/t20200309_214241. html (accessed Aug 16, 2020 )

2. Di L, Fu Y, Sun Y, et al. RNA sequencing by direct tagmentation of RNA/DNA hybrids. *Proc Natl Acad Sci U S A* 2020;**117**:2886-93.

3. Chen C, Li J, Di L, et al. MINERVA: a facile strategy for SARS-CoV-2 whole genome deep sequencing of clinical samples. *bioRxiv* 2020. <https://www.biorxiv.org/content/10.1101/2020.04.25.060947v2>

4. Xu Y, Kang L, Shen Z, et al. Hybrid capture-based sequencing enables unbiased recovery of SAR-CoV-2 genomes from fecal samples and characterization of the dynamics of intra-host variants. *bioRxiv* 2020. <https://www.biorxiv.org/content/10.1101/2020.07.30.230102v1>

5. Chen S, Zhou Y, Chen Y, Gu J. fastp: an ultra-fast all-in-one FASTQ preprocessor. *Bioinformatics* 2018; **34**:i884-i90.

6. Wood DE, Lu J, Langmead B. Improved metagenomic analysis with Kraken 2. *Genome Biol* 2019; **20**:257.

7. Li H. Aligning sequence reads, clone sequences and assembly contigs with BWA-MEM. 2013; arXiv:1303.3997. https://arxiv.org/abs/1303.3997.

8. Picard.http://broadinstitute.github.io/picard/.

9. Li H, Handsaker B, Wysoker A, et al. The Sequence Alignment/Map format and SAMtools. *Bioinformatics* 2009; **25**:2078-9.

10. Koboldt DC, Zhang Q, Larson DE, et al. VarScan 2: somatic mutation and copy number alteration discovery in cancer by exome sequencing. *Genome Res* 2012; **22**:568-76.

11. Kumar S, Stecher G, Li M, Knyaz C, Tamura K. MEGA X: Molecular Evolutionary Genetics Analysis across Computing Platforms. *Mol Biol Evol* 2018; **35**:1547-9.
